# The Impact of Coexisting Gestational Diabetes Mellitus on the Course of Preeclampsia

**DOI:** 10.3390/jcm11216390

**Published:** 2022-10-28

**Authors:** Katarzyna Pankiewicz, Ewa Szczerba, Anna Fijałkowska, Janusz Sierdziński, Tadeusz Issat, Tomasz Mikołaj Maciejewski

**Affiliations:** 1 Department of Obstetrics and Gynecology, Institute of Mother and Child in Warsaw, 01-211 Warsaw, Poland; 2 Department of Cardiology, Institute of Mother and Child in Warsaw, 01-211 Warsaw, Poland; 3 Department of Medical Informatics and Telemedicine, Medical University of Warsaw, Litewska 14/16, 00-581 Warsaw, Poland

**Keywords:** gestational diabetes mellitus, preeclampsia, soluble fms-like tyrosine kinase 1, placental growth factor

## Abstract

A strict correlation between gestational diabetes mellitus (GDM) and preeclampsia (PE) has been shown in previous studies. This case-control observational study evaluates the influence of concomitant GDM on the severity of PE. Ninety-nine patients were included: thirty-eight with PE without GDM (group 1), fourteen with PE and concomitant GDM (group 2), and forty-seven with uncomplicated pregnancies (group 3). Adverse maternal/fetal and neonatal outcomes were registered. Patients underwent blood sample analysis of serum PlGF, sFlt-1, creatinine levels, and platelet count (PLT). The incidence of preterm birth, FGR, HELLP syndrome, and NICU admission was significantly higher in group 1 in comparison to groups 2 and 3, whereas RDS was diagnosed most often in group 2 in comparison to groups 1 and 3. All studied biochemical parameters differed between the control group and both PE groups; however, there were no differences between patients with PE with and without GDM. The presented study indicates that the coexistence of GDM may mitigate the course of PE. The lack of differences between patients with PE with and without GDM in serum levels of studied biomarkers may also confirm its usefulness in the diagnosis and management of PE in patients with coexisting GDM.

## 1. Introduction

Preeclampsia (PE) complicates about 3–8% of pregnancies, whereas gestational diabetes mellitus (GDM) complicates about 8.7–14% of pregnancies [1,2,3]. PE is defined according to The International Society for the Study of Hypertension in Pregnancy (ISSHP) as the presence of a new-onset hypertension after 20 weeks’ gestation accompanied by proteinuria or evidence of maternal acute kidney injury, liver dysfunction, neurological features, hemolysis or thrombocytopenia, or fetal growth restriction (FGR) [4]. PE is a multifaceted disorder; however, an inadequate trophoblast invasion of maternal spiral arteries with subsequent maternal global endothelial dysfunction is seen as the main mechanism involved in the development of this disease [5]. During PE, maternal symptoms are a consequence of the imbalance between circulating angiogenic factors, i.a., vascular endothelial growth factor (VEGF), and placental growth factor (PlGF); and antiangiogenic factors, i.a., soluble fms-like tyrosine kinase 1 (sFlt-1) and soluble endoglin (sEng) [6]. Moreover, these biomarkers can be used in the screening and diagnosis of PE [7,8,9,10].

GDM is defined according to the International Diabetes Federation (IDF) as spontaneous hyperglycemia developing during pregnancy [11]. It is mostly the result of impaired glucose tolerance due to pancreatic β-cell dysfunction on a background of chronic insulin resistance. During normal gestation, women are able to counteract peripheral insulin resistance with a significant increase of their basal and nutrient-stimulated insulin secretion from pancreatic b cells. When women are not able to intensify insulin secretion, GDM occurs [12]. It is widely known that GDM is a risk factor for developing PE during pregnancy, and many studies have shown a strict correlation between these two diseases [13,14,15]. Additionally, both diseases may have serious long-term consequences for the mother and fetus, such as a significantly increased risk of developing hypertension, heart failure, stroke, end-stage chronic kidney disease (CKD), and diabetes mellitus later (DM) in life [16,17,18,19,20,21].

The risk of developing PE in patients with GDM has been, up to date, widely studied [22,23,24,25]. PE and GDM share several risk factors, such as advanced maternal age, nulliparity, multiple pregnancy, ethnicity, and pregestational obesity. Although both diseases also share some pathophysiological pathways (e.g., sterile inflammation), the main mechanisms take place in the placenta and present completely differently—proangiogenic state in GDM, and antiangiogenic state in PE [21]. This is a reason to suspect that the influence of GDM on the course of PE may be significant, causing a shift in the imbalance between proangiogenic and antiangiogenic factors derived from the placenta. On the other hand, there is also a possibility that the usefulness of the PE biomarkers PlGF and sFlt-1 in the diagnosis and prediction of pregnancy complications during PE might not be maintained on the same level in patients with both PE and GDM.

To our best knowledge, there is only one study referring to the impact of GDM on the course of pregnancy complicated by hypertensive disorders indicating an increased risk of adverse pregnancy outcomes in women with both gestational hypertension (GH) and GDM [26]. There is also another study comparing PE biomarkers in patients with PE alone, PE+GDM, GDM alone, and healthy controls demonstrating that sFlt-1 overproduction is also related to PE in GDM pregnancies, even though it is characterized by a less severe endothelial dysfunction [21].

The aim of this study was to evaluate the influence of concomitant GDM on the severity of PE and its complications (maternal and neonatal adverse outcomes), and the usefulness of PE biomarkers in patients with both PE and GDM. Both diseases are currently the most taxing and common problems in antenatal care, especially PE, being responsible for about 16–18% of maternal perinatal deaths and up to 40% of fetal and neonatal deaths [1]. Thus, after realizing the translational potential of this study, the better understanding of the coexistence of both diseases may lead to significant improvements in antenatal care.

## 2. Materials and Methods

### 2.1. Study Population and Protocol

In this case-control observational prospective study, adult women (>18 years of age) in singleton pregnancies admitted to the Department of Gynecology and Obstetrics, Institute of Mother and Child in Warsaw between November 2013–April 2018, with the diagnosis of preeclampsia with and without concomitant GDM were included. The results obtained in preeclamptic patients with and without GDM were compared to those obtained in healthy pregnant volunteers. PE was defined according to the 2011 European Society of Cardiology (ESC) Guidelines [27], whereas GDM was defined according to the IDF 2011 Guidelines [28]. The exclusion criteria for all groups included: gestational age < 22 weeks, multiple pregnancies, history of CKD, antiphospholipid syndrome, congenital and acquired heart defects, congenital or acquired coagulopathies (hemorrhagic diathesis or thrombophilia), pregestational diabetes, and symptoms of infectious diseases (including suspected chorioamnionitis). All patients underwent blood sample biochemical analysis—measurements of serum PlGF, sFlt-1, and creatinine levels, as well as platelet count (PLT). The following adverse maternal/fetal outcomes were evaluated: preterm birth, HELLP syndrome, FGR, oligohydramnion, and placental abruption. Additionally, the following adverse neonatal outcomes were registered: admission to neonatal intensive care unit (NICU), respiratory distress syndrome (RDS), necrotic enterocolitis (NEC), intraventricular hemorrhage grade III and IV (IVH III and IV), sepsis, bronchopulmonary dysplasia (BPD), and neonatal death. HELLP syndrome was defined as elevated liver enzymes (ASPAT > 70 U/L), hemolysis (LDH > 600 U/L), and low platelets (<100,000/mL). FGR was defined as an estimated intrauterine weight below the 10th percentile after gestational age had been confirmed by a first-trimester ultrasound. Oligohydramnion was diagnosed when the amniotic fluid index (AFI) was below the 5th percentile for the gestational age.

The study was approved by the Local Bioethics Committee at the Institute of Mother and Child, and written informed consent was obtained from all participants. The study was performed in accordance with the guidelines described in the Declaration of Helsinki [29].

### 2.2. Blood Sample Preparation and Analysis

Whole blood samples were collected on the day of enrollment to the study, then were centrifugated at 25 °C for 20 min at 2000× *g*, and obtained sera were stored at −80 °C until further analysis. Serum sFlt-1 and PlGF levels were assessed using fully automated immunoassays (Elecsys^®^ sFlt-1 and Elecsys^®^ PlGF, Roche Diagnostics, Germany), and then the sFlt-1/PlGF ratio was calculated. Serum creatinine levels were assessed using automated kinetic colorimetric assay based on the Jaffé method in an alkaline solution, with picrate (Creatinine Jaffé Gen.2^®^, Roche Diagnostics, Germany). The estimated glomerular filtration rate (eGFR) was calculated using the Chronic Kidney Disease Epidemiology Collaboration (CKD-EPI) equation (gender- and race-specific): GFR = 141 × min (Scr/κ, 1)^−0.329^ × max (Scr/0.7, 1)^−1.209^ × 0.993^age^ × 1.018, where Scr is a serum creatinine level. The gold standard in GFR estimation is 24 h urine creatinine clearance, but it can be troublesome in different circumstances. The authors are aware that none of the available GFR formulas were fully validated in pregnancy; however, it is only additional information in this study, and the authors treat it with caution.

### 2.3. Statistical Analysis

The survey was conducted on the basis of strictly prepared forms. The dataset was collected in a relational database. The collected research material was analyzed using SAS 9.4 statistical software (SAS Institute Inc., 100 SAS Campus Drive, Cary, NC 27513-2414, USA). Continuous variables are expressed as the mean +/− SD and median, with a sample representativeness of 95% CI. Discrete variables are presented as numbers or letters, and categorical variables are marked accordingly. Statistical analysis describing the interrelationships between the examined variables, as well as comparisons between patients groups, were performed using the Mann–Whitney U test, Chisq test, and ANOVA Kruskal–Wallis. The value of *p* < 0.05 was taken as the significance level of the above-mentioned analyses.

## 3. Results

Ninety-nine patients were included in the study: thirty-eight with PE without GDM (group 1), fourteen with PE and concomitant GDM (group 2), and forty-seven with uncomplicated pregnancies (group 3). In the analyzed time period, our department carried out about 1700 deliveries, and among them, about 20–22 patients were diagnosed with PE, including about 4–5 women with PE and GDM. Hence, the vast majority of the PE population hospitalized in our clinic was enrolled (a few patients did not agree to participate in the study and could not be included).

Among patients with PE and GDM, 11 (78.57%) women were treated with diet alone (GDMG1), and 3 (21.43%) were treated with insulin (GDMG2). The demographic and clinical characteristics of the study participants are presented in Table 1. One of the most important differences between the groups was the mode of delivery—the rate of cesarean section was highest in group 2 in comparison to group 1 and 3.

### 3.1. Maternal/Fetal Outcomes

**Figure 1:**
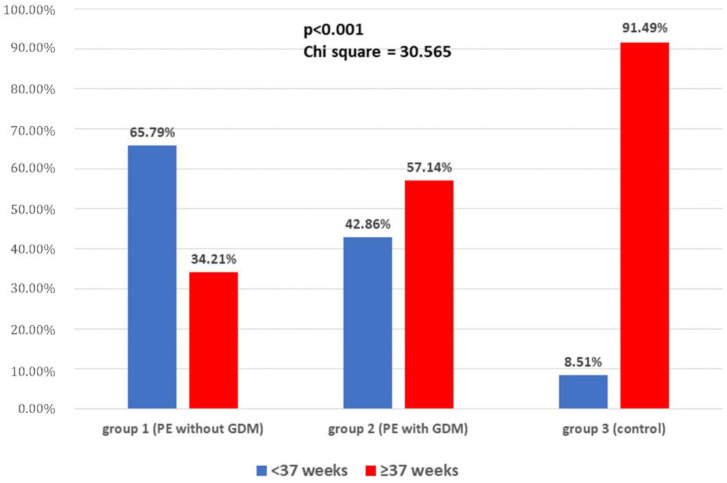
The incidence of preterm delivery in the study groups.

The incidence of preterm birth was highest in group 1, with 25 (65.79%) cases, whereas in group 2, it was 6 (42.86%) cases, and 4 (8.51%) in group 3. All these differences were statistically significant (Chi^2^ = 30.56, *p* < 0.001; Figure 1). Similarly, FGR was diagnosed most often in group 1: 18 (47.37%) patients, in comparison to 2 (14.29%) cases in group 2, and 1 (2.13%) patient in the control group (Chi^2^ = 26.2, *p* < 0.001). HELLP syndrome occurred in seven (18.42%) patients with PE without GDM, and there was no case of this complication in other groups (Chi^2^ = 12.09, *p* = 0.002). There was no difference between groups in the incidence of oligohydramnion (Chi^2^ = 0.92, *p* = 0.63). Considering the intensiveness of antihypertensive treatment, there was a significant difference between group 1 and 2. Monotherapy was effective in 14 (36.84%) patients in group 1, and 7 (50%) women in group 2 (Chi^2^ = 25.15, *p* < 0.001), whereas polytherapy with two or more drugs was necessary in 24 (63.16%) patients in group 1, and 7 (50%) patients in group 2 (Chi^2^ = 41.62, *p* < 0.001). All preeclamptic patients (with and without GDM) received methyldopa as a first-choice treatment. When it was insufficient for appropriate blood pressure control, patients were treated with either beta blockers (labetalol or metoprolol) or calcium antagonists (amlodipine or nifedipine). In group 1, 23 (60.5%) patients received a beta blocker, and 11 (28.9%) received a calcium antagonist, whereas in group 2, 7 (50%; Chi^2^ = 0.46, *p* =0.5) women were treated with beta blockers, and 4 (28.6%; Chi^2^ = 0.0007, *p* = 0.98) with calcium antagonists. There was also a need for magnesium sulfate (MgSO_4_) infusion in six (15.79%) patients with PE alone, and in three (21.43%; Chi^2^= 0.2273, *p* = 0.63) patients with PE and GDM.

### 3.2. Neonatal Outcomes

There were significant between-group differences in neonatal outcomes. The mean birthweight was significantly higher in the control group (3273.5 ± 491.56 g; median 3300 g, 95% CI 3129.184–3417.84 g) than in the PE groups, both without and with GDM (2239.2 ± 762.58 g, median 2220 g, 95% CI 1988.56–2489.86 g; *p* < 0.001; and 2523.6 ± 1079.3 g, median 2485 g, 95% CI 1900.4–3146,74 g; *p* = 0.043, respectively). However, there was no difference in the mean birthweight between group 1 and 2 (*p* = 0.461). The first-minute Apgar score was significantly higher in the control group than in group 1 (9.64 vs. 8.76; *p* = 0.028), but there were no differences between all groups in the fifth-minute Apgar score. Among all 99 newborns included in the study, one died (in group 2). The admission to NICU was necessary in eight (21.05%) babies in group 1, and two (14.29%) newborns in group 2, whereas none of the children born in the control group needed NICU admission. These differences were statistically significant (Chi^2^ = 10.57, *p* = 0.005). Among other neonatal complications, RDS was diagnosed most often in group 2: five (35.71%) cases in comparison to six (15.79%) cases in group 1, and one (2.13%) case in the control group (Chi^2^ = 12.20, *p* = 0.002). Neonatal outcomes are presented in detail in Table 2.

### 3.3. Biochemical Parameters

In all studied biochemical parameters and ratios, i.e., serum PlGF, sFlt-1, and creatinine levels, eGFR and PLT differed between the control group and both PE groups; however, there were no differences in all these parameters between patients with PE with and without GDM. These results are presented in Table 3.

## 4. Discussion

The present study aimed to assess the possible impact of GDM coexistence on the course and severity of PE. We demonstrated that women with PE and GDM were less likely to give birth prematurely and to develop FGR and HELLP syndrome, and required less intensive antihypertensive treatment than women with PE, but without GDM. The cesarean section rate was, in turn, significantly higher in women with both diseases than with PE alone. Newborns from mothers with PE and GDM less frequently required admission to the NICU; however, they were more likely to develop RDS than newborns from mothers with PE without GDM.

Available studies demonstrated a strong association between diabetes and PE, i.e., both pregestational and GDM were confirmed as risk factors for developing PE. PE is diagnosed in 15–20% of pregnant women with type 1 diabetes and in 10–14% of pregnant women with type 2 diabetes in comparison to 2–8% of women without diabetes [30,31]. In a study based on the German Perinatal Quality Registry including 647,392 patients, the risk of developing PE was increased in patients with GDM, even after adjustment by age, nationality, job status, smoking, parity, multiple pregnancy, pre-pregnancy weight, and gestational weight gain (OR 1.29, 95% CI 1.19–1.41) [13]. Moreover, other birth registry studies, performed in Sweden and Canada, confirmed GDM as an independent risk factor for PE [14,32]. Additionally, women with PE in their first pregnancy have an increased risk of GDM in the second pregnancy in comparison to patients with neither of these two diseases in their first pregnancy (OR 1.2, 95% CI 1.1–1.3) [22]. The association of GDM and PE may be related to the common risk factors shared by both diseases, such as advanced maternal age, nulliparity, multiple pregnancies, obesity, and Black race. However, many maladaptations to pregnancy present in both diseases have been also identified. They include insulin resistance, endothelial dysfunction, angiogenic imbalance, and oxidative stress [15,33,34]. Recent studies demonstrated that other signaling pathways may also be involved in the pathophysiology of PE in GDM patients, e.g., kisspeptin-1 and its receptor [35]. The genetic background makes it possible to underline the association between GDM and PE as well—to date, ACE gene I/D polymorphism and miRNA146A rs2910164 (G/C) polymorphism have been confirmed as being related to an increased incidence of PE in women with GDM [25,36].

Both PE and GDM are related to an increased risk of adverse perinatal outcomes, and, thus, are important global public health concerns. Nunes et al. investigated the influence of PE, advanced maternal age, and maternal obesity on neonatal outcomes in patients with GDM. They found that among these three factors, only the coexistence of PE showed an association with adverse neonatal outcomes, such as neonatal morbidity, low and very low birthweight, and preterm delivery [24]. To our knowledge, there is only one study comparing perinatal outcomes in patients with hypertensive disorders of pregnancy (HDP) with and without GDM. Preterm delivery rates in this study were more than threefold greater in the HDP and GDM group and HDP-alone group in comparison to healthy controls, with an adjusted OR of 4.84 (95% CI 4.34–5.4) and 3.92 (95% CI 3.65–4.21), respectively. Additionally, the rate of small-for-gestational-age (SGA) babies was greater in patients with HDP with and without GDM in comparison to the control group, with an adjusted OR of 6.57 (95% CI 5.56–7.75) and 5.81 (95% CI 5.15–6.55), respectively. The incidence of adverse outcomes increased further in women with PE and eclampsia [26]. Our results contradict these results, because in our research, the incidence of preterm birth and FGR was lower in patients with PE and coexisting GDM than in women with PE alone. Our results indicate the less severe course of PE in women with concomitant GDM, as evidenced also by a lower incidence of NICU admissions among neonates from mothers with PE and GDM than in PE alone. On the other hand, there was an increased rate of RDS among babies from mothers suffering from both PE and GDM, but there is a vast body of evidence that GDM alone is associated with an increased risk of neonatal RDS [37], because maternal hyperglycemia delays fetal lung maturation [38].

Another important finding of the presented study is that the cesarean section rate was highest in patients with both PE and GDM, and this is consistent with earlier studies, including the above-mentioned research performed by Lin et al. [26]. GDM is a common risk of ending delivery with C-section, which is also associated with fetal macrosomia observed among women with GDM [39,40].

As mentioned at the beginning, the main mechanism of developing PE is thought to be an imbalance between angiogenic and antiangiogenic factors leading to maternal global endothelial dysfunction. In our study, we compared the serum levels of the most important angio- and antiangiogenic biomarkers, i.e., PlGF and sFlt-1, in patients with PE with and without coexisting GDM. As expected, significant differences were found between women with PE (both with and without GDM) and healthy pregnant women. Nevertheless, there were no statistically significant differences between patients with PE and GDM and PE alone. We suggest that such a result may indicate no impact of GDM on the usefulness of these biomarkers in clinical practice to predict PE and related adverse outcomes. Similar to our study, Nuzzo et al. found that the serum sFlt-1 level was significantly increased and serum PlGF was significantly decreased in patients with PE alone and PE with GDM in comparison to women with GDM alone and healthy controls. However, they also demonstrated higher values of sFlt-1/PlGF ratio in patients with PE alone than in PE and GDM. Clinical parameters, such as the incidence of pathological umbilical Doppler, low Apgar score, and NICU admission, were, in this study, increased in women with PE alone in comparison with PE-GDM patients, suggesting the possible influence of GDM on the course of PE, which is consistent with our study as well [41]. Cohen et al. presented elevated serum levels of sFlt-1 and reduced serum levels of PlGF in patients with pregestational diabetes who developed PE, just as women without diabetes have been shown to have in PE [42]. Kapustin et al. investigated the placental expression of PlGF and endoglin in patients with PE and GDM, and found that PlGF expression was undermost in PE. In GDM treated with insulin, PlGF expression was also reduced in comparison to GDM patients treated only with diet, indicating that placental PlGF expression in GDM is also altered and dependent on the control of the glycemia level during pregnancy [43]. There is evidence that in women with obesity, GDM may modify the association between PlGF in early pregnancy and PE [44]. Nonetheless, in our study, there was no difference between the incidence of obesity in all studied groups; thus, there is no risk of bias in this point.

Less is known about the mechanism that could be a reason of the possible phenomenon that GDM might alleviate the course of PE. However, in a recently published study, Kul et al. investigated the prevalence of coronary microvascular dysfunction (CMD) in women with combined PE and GDM. They found that in patients with GDM, additional PE on top of GDM was associated with a significant increased risk of CMD, even after adjusting for other covariates, but for patients with PE, the presence of GDM did not confer an additional risk after multivariate analysis [21]. The authors of this study suggest two possible explanations. First, is that PE has a direct and immediate effect on vasculature independent from hypertension, whereas the influence of disturbed glucose metabolism is time-dependent [21]. The second thing might be the level of immune activation, which is one of the central parts in the pathogenesis of PE, as well as endothelial dysfunction and CMD, whereas in GDM, the evidence for the role of inflammation in the pathophysiology of the disease is less robust [45,46]. Pro-inflammatory cytokines secreted in adipose tissue in GDM (such as TNFα, IL-1β, IL-6) impair insulin signaling and inhibit insulin release from β-cells; however, this relationship is complex and not straightforward, because there are studies reporting that GDM placentae secrete fewer pro-inflammatory cytokines than healthy placentae [12]. The difference in the immune response in PE and GDM should also be considered as one of the important mechanisms participating in the described PE and GDM coexistence phenomenon.

The potential mitigation of the severity of PE by the coexisting GDM has its justification in the pathophysiology of both diseases. PE is well-documented as an antiangiogenic state with general vasoconstriction and reduced placental perfusion. GDM is, in turn, considered as a pro-angiogenic state, and diabetic placentas exhibit enhanced vascularization in comparison to placentas from uncomplicated pregnancies. These results appear to be related to the reduced expression of Flt-1, and the increased activity of VEGF receptor 2 (KDR) [47]. The change in the placental VEGF/VEGF receptor expression ratio in hyperglycemia may favor angiogenesis in placental tissue, and could explain the hypercapillarization of villi seen in diabetic patients [48]. In our study, it is reflected in the incidence of FGR that was higher in PE patients without GDM. Increased placental weight and a decreased percentage of pathological uterine/umbilical Doppler in PE-GDM patients in comparison to PE alone in the above-mentioned study performed by Nuzzo et al. can also provide evidence of the possible placental adaptation attempt [41].

Another possibility is the potential impact of differences in the metabolic profiles of patients with and without GDM, and the incidence of metabolic syndrome (MetS) among them. MetS is a cluster of cardiovascular disease risk factors, including obesity, atherogenic dyslipidemia, raised blood pressure, insulin resistance, and pro-inflammatory states; although, there are several definitions and cut-points to describe and characterize MetS [49,50]. Women with MetS are at an increased risk of both PE and GDM [51,52]. Moreover, Grieger et al. demonstrated that more than half of women who had MetS in early pregnancy developed a pregnancy complication, in comparison to just over a third of women who did not have MetS [51]. We have no data about the components of MetS in our cohort; however, we can hypothesize that the results in patients with PE and GDM may arise more from metabolic abnormalities than from enhanced endothelial dysfunction, as in “pure” PE, because of different pathophysiological pathways, similar to the difference between early- and late-onset PE. The risk factors for cardiovascular disorders and PE are very similar, and it still remains unclear whether PE is an individual risk factor for future cardiovascular events or an early marker of women with high-risk profiles for cardiovascular disease, where the pregnancy can only be a trigger for cardiovascular alterations that manifest in the development of PE [20,53]. This leaves a lot of room for further research to explain these dependencies.

The main limitation of this study is the relatively small number of cases; thus, further analyses are required to confirm our data. The lack of a group of patients with GDM alone may also be considered as a limitation, because it could provide more important information. However, the authors recognized both diseases as having completely different pathophysiology and perinatal/neonatal outcomes (e.g., FGR vs. macrosomia), and focused on PE and the possible effect of coexisting GDM on the course of it. Moreover, the research discussed earlier in this paper presented different outcomes: in the GDM-alone group, the patients came closer to the control group than to PE patients [24,41].

The strength of the study is its prospective design and the high group homogeneity obtained, i.a., by applying narrow inclusion criteria (proteinuria as a necessary component), and the exclusion of multiple pregnancies, pregestational diabetes, and preexisting hypertension. In our opinion, proteinuria as a necessary component is an advantage, because it increases group homogeneity and helps avoiding bias related to the differences between many individual components of new PE definition (such as liver dysfunction, neurological features, thrombocytopenia, or FGR). There are only few data concerning the relationship between these components with the severity of the imbalance between angio- and antiangiogenic factors [6,54,55,56]; our previous study may serve as an example, where we demonstrated that in preeclamptic patients, renal function parameters correlate with serum sFlt-1 levels and sFlt/PlGF-1 ratio [57].

The strength of the study is also its translational potential—improving the knowledge of the pathomechanisms underlying the coexistence of PE and GDM, especially the molecular basis of the possible alleviating effect of GDM on the course of PE, which may lead to elaborating tailored, successful preventive and therapeutic strategies for women at a high risk of developing PE and its severe complications.

## 5. Conclusions

The presented study demonstrated that the incidence of adverse perinatal outcomes, such as preterm birth, FGR, HELLP syndrome, and neonatal admission to NICU, was significantly lower among women with PE and GDM in comparison to patients with PE alone, indicating that the coexistence of GDM may mitigate the course of PE. Additionally, there was no difference between patients with PE with and without GDM in the serum levels of biomarkers, such as sFlt-1 and PlGF, as well as in sFlt-1/PlGF ratio, confirming no influence of GDM on its usefulness in the diagnosis and management of PE.

## Figures and Tables

**Table 1:** Demographic and clinical characteristic of study participants.

	Group 1 (n = 38) PE without GDM	Group 2 (n = 14) PE with GDM	Group 3 (n = 47) Control Group	Chi^2^	*p* Value
Maternal age (years) Gestational age at study enrollement (weeks) Gestational age at delivery (weeks)	31.53 ± 5.68 33.58 ± 2.85 34.97 ± 2.95	34.93 ± 3.29 33.57 ± 4.33 34.64 ± 4.38	30.4 ± 4.9 36.49 ± 2.24 38.53 ± 1.73	N/A N/A N/A	† †† ‡
Nulliparity	27 (71.05%)	7 (50.0%)	24 (51.06%)	3.96	0.48
Maternal Medical history: Hypothyroidism and other concomitant disease *	14 (36.84%)	8 (57.14%)	13 (27.7%)	4.16	0.125
Obesity	12 (31.58%)	7 (50.0%)	11 (23.4%)	3.66	0.16
Delivery mode: Cesarean section Vaginal birth	31 (81.58%) 7 (18.42%)	13 (92.86%) 1 (7.14%)	12 (25.53%) 35 (74.47%)	35.61 33.32	<0.001 <0.001

† Kruskal–Wallis test *p* value gr. 1 vs. 2 = 0.066, gr. 1 vs. 3 = 0.99, gr. 2 vs. 3 = 0.007; †† Kruskal–Wallis test *p* value gr. 1 vs. 2 = 1.00, gr. 1 vs. 3 < 0.01, gr. 2 vs. 3 = 0.027; ‡ Kruskal–Wallis test *p* value gr. 1 vs. 2 = 1.00, gr. 1 vs. 3 < 0.01, gr. 2 vs. 3 < 0.01. * other concomitant diseases in patients included in the study were: asthma, sarcoidosis, ulcerative colitis, juvenile arthritis, rheumatoid arthritis; these data were collected from patients’ medical history and not from personal examination; thus, diagnostic criteria could differ between individuals; PE, preeclampsia; GDM, gestational diabetes mellitus; Chi^2^, chi-squared test; N/A, not applicable.

**Table 2:** Neonatal outcomes.

	Group 1 (n = 38) PE without GDM	Group 2 (n = 14) PE with GDM	Group 3 (n = 47) Control Group	Chi^2^	*p*
Birthweight (g)	2239.2 ± 762.58	2523.6 ± 1079.3	3273.5 ± 491.56	N/A	†
First-minute Apgar score	8.76 ± 1.62	8.29 ± 2.49	9.64 ± 0.965	N/A	††
Fifth-minute Apgar score	9.54 ± 0.77	9.29 ± 1.38	9.89 ± 0.43	N/A	‡
Neonatal complicationsDeath	0	1 (7.14%)	0	6.13	0.047
Admission to NICU	8 (21.05%)	2 (14.29%)	0	10.57	0.005
RDS	6 (15.79%)	5 (35.71%)	1 (2.13%)	12.20	0.002
IVH III/IV	1 (2.63%)	0	0	1.62	0.445
NEC	2 (5.26%)	0	0	3.28	0.194
sepsis	3 (7.89%)	0	0	4.97	0.084
BPD	2 (5.26%)	0	0	3.28	0.194
without any complication	30 (78.95%)	8 (57.14%)	46 (97.87%)	15.59	<0.001

† Kruskal–Wallis test *p* value gr. 1 vs. 2 = 0.461, gr. 1 vs. 3 = < 0.001, gr. 2 vs. 3 = 0.043; †† Kruskal–Wallis test *p* value gr. 1 vs. 2 = 1.0, gr. 1 vs. 3 = 0.028, gr. 2 vs. 3 = 0.052; ‡ Kruskal–Wallis test p value gr. 1 vs. 2 = 1.0, gr. 1 vs. 3 = 0.14, gr. 2 vs. 3 = 0.29; PE, preeclampsia; GDM, gestational diabetes mellitus; NICU, neonatal intensive care unit; RDS, respiratory distress syndrome; IVH III/IV, intraventricular hemorrhage grade III or IV; NEC, necrotic enterocolitis; BPD, bronchopulmonary dysplasia; Chi^2^, chi-squared test; N/A, not applicable.

**Table 3:** Biochemical parameters (median value of each parameter with SD).

	Group 1 (PE without GDM)	Group 2 (PE with GDM)	Group 3 (Control)	*p* Value (Kruskal–Wallis Test)		
				Gr. 1 vs. 2	Gr. 1 vs. 3	Gr. 2 vs. 3
sFlt-1 (pg/mL)	12054.0 ± 6757.45 (95% CI 10842.65–15284.88)	10109.5 ± 5265.4 (95% CI 7966.46–14046.8)	3556.0 ± 2218.7 (95% CI 3160.69–4463.56)	1.00	<0.001	<0.001
PlGF (pg/mL)	54.75 ± 75.33 (95% CI 50.46–109.98)	68.70 ± 53.38 (95% CI 52.47–114.1)	192.9 ± 404.7 (95% CI 209.66–447.32)	1.00	<0.001	<0.001
sFlt-1/PlGF	209.27 ± 253.88 (95% CI 189.73–356.63)	162.7 ± 327.37 (95% CI 66.9–444.9)	15.7 ± 22.24 (95% CI 16.97–30.02)	1.00	<0.001	<0.001
PLT	189.5 ± 58.88 (95% CI 174.88–213.59)	159.0 ± 79.6 (95% CI 136.7–228.7)	217.0 ± 49.69 (95% CI 211.54–240.72)	0.868	0.022	0.008
Creatinine (µmo/L)	55.0 ± 12.71 (95% CI 53.03–61.63)	61.0 ± 15.88 (95% CI 53.05–71.38)	49.0 ± 7.09 (95% CI 45.17–50.04)	1.00	<0.01	0.001
eGFR (mL/min/1.73^2^)	119.8 ± 15.92 (95% CI 110.03–120.81)	112.05 ± 18.85 (95% CI 95.78–117.55)	124.6 ± 8.24 (95% CI 123.74–129.4)	0.487	0.004	<0.001

PE, preeclampsia; GDM, gestational diabetes mellitus; sFlt-1, soluble fms-like tyrosine kinase; PlGF, placental growth factor; PLT, platelet count per mm^3^; eGFR, estimated glomerular filtration rate.

## Data Availability

Not applicable.
